# Avoiding the Interference of Doxorubicin with MTT Measurements on the MCF-7 Breast Cancer Cell Line

**DOI:** 10.3390/mps2020029

**Published:** 2019-04-12

**Authors:** Carla Luis, Yuselis Castaño-Guerrero, Raquel Soares, Goreti Sales, Rúben Fernandes

**Affiliations:** 1 School of Health, Polytechnic Institute of Porto, 4200 Porto, Portugal; karlaluiz@hotmail.com; 2 Institute of Research and Innovation in Health (i3S), Porto University, 4200 Porto, Portugal; raqsoa@med.up.pt; 3 Faculty of Medicine, Porto University, 4200 Porto, Portugal; 4 BioMark-CEB/ISEP, School of Engineering, Polytechnic Institute of Porto, 4200 Porto, Portugal; ycastano87@gmail.com (Y.C.-G.); goreti.sales@gmail.com (G.S.)

**Keywords:** MTT, doxorubicin, chemotherapy, breast cancer, MCF-7

## Abstract

Doxorubicin (DOXO) is an adjuvant chemotherapy agent and is also commonly used in cell biology research. Cytotoxic assays in cell culture are frequently used in order to stablish drug concentrations that are useful for controlling cell proliferation. One common cytotoxic method used is 3-(4,5-Dimethylthiazol-2-yl)-2,5-Diphenyltetrazolium Bromide (MTT). Our present research aims to support future studies in engaging MTT assay using DOXO that exhibits a strong red coloration and fluorescence, and so it is assumed that DOXO may interfere with commonly used colorimetric assays such as MTT. The interference of DOXO in the MTT determination was evaluated in a Breast Cancer cell line Michigan Cancer Foundation-7 (MCF-7). The interference was evaluated by means of spectroscopic methods in particular spectrophometry and fluorescence spectroscopy of MTT and DOXO. We postulate that the medium and the MTT reagent itself can interfere on the metabolic activity method, so in order to achieve better results, DMEM was replaced by a neutral buffer like Phosphate-buffered saline (PBS). This protocol may be extremely useful in future studies involving DOXO.

## 1. Introduction

Breast cancer (BrCa) is the most common cancer among women and although advances in screening and treatment have improved, it is still the most invasive form of cancer. Currently, the first line treatments against BrCa are surgery with radiotherapy, supplemented with chemotherapy. Doxorubicin (DOXO) is used as an adjuvant therapy component in women with evidence of axillary lymph node involvement, following resection of primary BrCa [1].

DOXO is the leading compound of a wide family of pharmaceutical anticancer anthracyclines first discovered from actinobacteria *Streptomyces peucetius* in the 1960s. It is a DNA intercalator that inhibits topoisomerase I and II, causing DNA damage and formation of reactive oxygen species (ROS) that promote caspases activation, and therefore, leads to apoptosis [2]. Frequently used alone, it can also be administrated in combination with other pharmaceutical agents to treat several types of malignancies including sarcoma, lymphoma, leukemia and breast cancer.

In order to better understand the mechanisms underlining the analysis of DOXO in cell viability, we used a breast cancer cell line (MCF-7) that requests Dulbecco’s Modified Eagle Medium (DMEM). Previous studies demonstrated that the highest interference in absorbance reading of DMEM medium is the pH indicator, phenol red [3]. Phenol red color exhibits a gradual transition from yellow (λ = 443 nm) to red (λ = 570 nm) and can be an interference in colorimetric DOXO assays.

Another potential interference in DOXO is the 3-(4,5-Dimethylthiazol-2-yl)-2,5-Diphenyltetrazolium Bromide (MTT) reagent which is soluble in culture medium and is cell permeable. MTT measures metabolic activity since it is mostly based on the cleavage of the tetrazolium ring in active mitochondria, thus the reaction occurs only in living cells (3). For this reason, it is commonly used to determine cell proliferation or cytotoxicity of drugs. MTT is reduced to formazan by a variety of intracellular oxidoreductase enzymes, most notably Nicotinamide adenine dinucleotide (NADH) reductase, and these formazan crystals can be quantified by absorbance measures within λ = 500 and λ = 600 nm wavelengths [4]. Thus, formazan absorption spectra overlap with the absorption spectra of DMEM/phenol red and with DOXO.

Since DOXO, Phenol Red containing medium DMEM and formazan crystals are emitting in visible light, we hypothesize that they are involved in interference. Therefore, the aim of the present study was to determine whether DOXO interferes with MTT and pH indicators containing medium.

## 2. Materials and Methods

### 2.1. Materials

#### 2.1.1. Chemicals

Phosphate Buffered Saline, or PBS (Amresco, Solon, OH, USA), Doxorubicin hydrochloride (DOXO, Sigma Aldrich, St. Louis, MO, USA). Aqueous stock solutions of DOXO was prepared using 18.2 MΩ ultrapure water obtained from a Milli-Q Plus Millipore water filtration system; Dulbecco’s Modified Eagle Medium (DMEM) (Sigma, St. Louis, MO, USA); Fetal Bovine Serum (FBS) (Invitrogen Life technologies, Carlsbad, CA, USA); penicillin/streptomycin (Invitrogen Life technologies, Carlsbad, CA, USA); Sodium bicarbonate (Merck, Darmstadt, Germany); Dimethyl Sulfoxide (DMSO, Merck, Darmstadt, Germany), MTT reagent (Abcam, Cambridge, UK).

#### 2.1.2. Cells 

Human breast carcinoma cells (MCF-7) was obtained from Professor Raquel Soares (Biomedicine department, Faculty of Medicine, University of Porto, Porto, Portugal).

#### 2.1.3. Equipment

UV−Vis spectrophotometer (Evolution 220); Fluorescence spectrophotometer (F-4500); Humidified atmosphere chamber; Inverted microscope, Neubauer Chamber Cell Counting. 

### 2.2. Methods

#### 2.2.1. Absorbance Spectra 

An absorbance scanning was performed using a UV-VIS spectrophotometer from 450 to 650 nm wavelength of MTT, formazan, DMEM, MTT, DMEM+MTT at the final concentration used in the assay.

#### 2.2.2. Intrinsic Emission Fluorescence by Fluorescence Spectrometry

The fluorescence spectra of DOXO standard were analyzed using a fluorescence spectrophotometer. Spectra were recorded on a 1 cm path-length cuvette with an excitation slit width of 20 nm and an emission slit width of 20 nm. The excitation wavelength was set at 480 nm and the emission spectra were recorded starting at 500 nm, ending on 700 nm.

#### 2.2.3. Dose-Response Curve Based on MTT

Human breast carcinoma cells (MCF-7) were cultured in DMEM, supplemented with 10% FBS, 1% penicillin/streptomycin and 3.7 g/L sodium bicarbonate and maintained at 37 °C in a humidified atmosphere containing 5% CO_2_. The cells were used between passages 17 to 29. 

MCF-7 cells were seeded in a 96-well plate at a density of 1 × 10^5^ cells/mL and incubate in complete medium at 37 °C. Treatment was administered after 24 h. DOXO was formulated in serum free medium in concentrations of 0.05, 0.1, 0.2, 0.4, 0.8, 1, 1.6 and 3 μg/mL, and 3 replicates were used for each concentration, with a final volume of 200 μL. Following 24 h of treatment incubation, the medium was removed and changed to PBS, 20 μL of 20 mM of MTT (dissolved in PBS) was added and incubated for 3 h at 37 °C. After incubation, the solution was carefully removed and 200 μL DMSO was added in order to solubilize the violet formazan crystals. The plates were then shaken on a horizontal shaker for 5 min to allow for complete dissolution. The optical density (OD) was read using Multiskan Ascent spectrophotometer (ThermoFischer, Waltham, MA, USA) at a dual wavelength of λ = 550/650 nm. Analyses were performed in triplicate. 

## 3. Results

### 3.1. Absorbance Spectra

**Figure 1:**
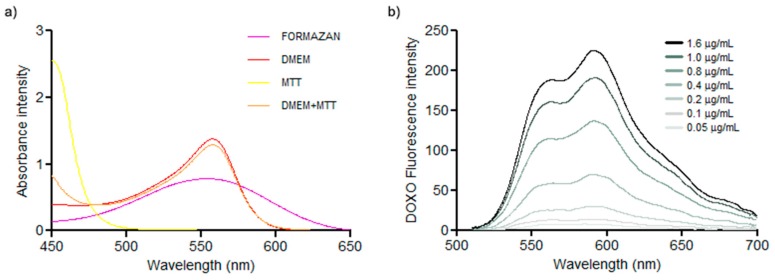
Comparison of the absorbance spectra of all intervenient compounds in MTT assay. Absorbance scanning between 450 and 650 nm wavelength where it is possible to see the overlapping of DMEM, Formazan and DMEM+MTT at 560 nm (**a**). The fluorescence spectra of DOXO standard with excitation wavelength set to 480 nm and emission spectra recorded starting in 500 nm, ending on 700 nm, once again, a peak at 560 nm coincident to the measurements of formazan, DMEM and MTT. It is also possible to observe a correlation between concentration and fluorescence intensity (**b**).

When comparing the absorbance of DOXO, MTT, Formazan, DMEM and DMEM+MTT it is possible to observe that there is a superposition between DOXO, Formazan, DMEM and DMEM+MTT (Figure 1) that can directly interfere in colorimetric methods. Moreover, the fluorescence spectrum of DOXO characteristically presents two main peaks when dissolved in water (at λ = 560 nm and at λ = 593 nm), the first peak is also overlaid with the other compounds.

### 3.2. Dose-Response Curve Based on MTT

**Figure 2:**
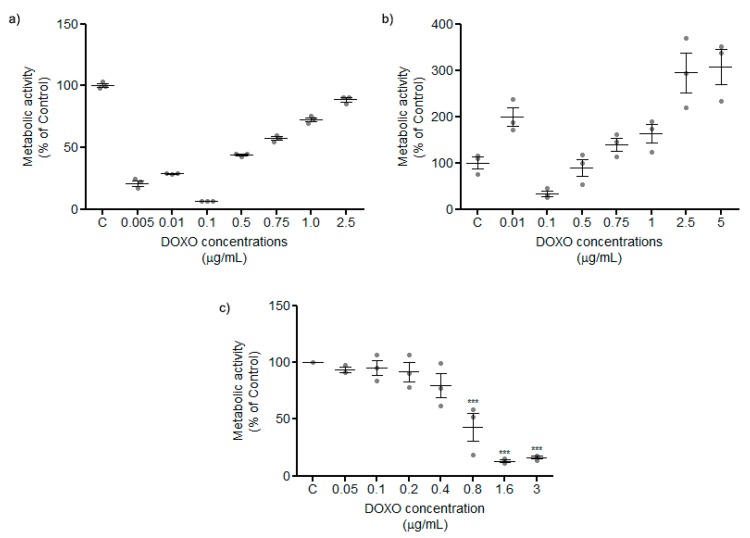
(**a**) and (**b**) Representations of metabolic activity by MTT incubated with DMEM medium at 12 and 24 h, respectively, in different DOXO concentrations. In both cases, results are not acceptable for high DOXO concentrations that reveal high viability rates (**c**) Representation of cytotoxicity of different concentrations of DOXO after wash and incubation of MTT reagent with PBS. With our protocol, the results become acceptable because they described an expected dose-response curve.

Using a protocol of MTT accordingly to the manufacturer’s instructions with 12 h (Figure 2a) and 24 h (Figure 2b) incubation periods with different DOXO concentrations on the MCF-7 cell line, with MTT reagent added directly to the medium, a tendency to increase the metabolic activity was observed, which contradicted the expected cytotoxic action of this drug.

In order to address this issue, DMEM was changed to PBS and then MTT assay was conducted according to the manufacturer’s instructions. Changing DMEM to PBS, nonsense plots disappear, and the result plot is an expected dose-response curve where the metabolic activity of cancer cells decreases in a constant manner (Figure 2c).

## 4. Discussion and Conclusions

Analyzing the spectra of the reagents of MTT protocol individually, absorbance of MTT itself does not overlap the absorbance spectrum neither with DOXO nor formazan. However, both DMEM and the mixture DMEM-MTT have their highest absorbance at the same wavelength as DOXO and formazan crystals (Figure 1). This must to be taken into consideration when using other colorimetric methods involving cell culture and DOXO in the sense that such overlap will interfere with each other. Regarding DOXO spectra it also interferes with MTT, probably due to the incubation time in the DMEM/MTT period. When MTT is incubated in PBS, this interference disappears since DMEM was wash out of the reading plate well. 

Analysis of all spectra was crucial for the understanding that, although DMSO is considered the best solvent for dissolving formazan crystals [5], residual medium/treatments can still be found in the microtiter wells and can indeed change the shape of the absorbance spectra.

MTT is still amongst the most common methods used to determine proliferation, viability, toxicity and metabolic activity, although an emerging understanding indicates many limitations of this method regarding complexity, sensitivity and retrieval [6]. We recognized that another limitation must be taken in account: reactions with the colorimetric nature of the compounds in study in each particular experimental setup. 

Taking DOXO as an example, every single colored compound whose effect on cells is under the assessment of a cytotoxicity assay using dyes such as formazan might interfere with each other. DOXO spectra overlaps with formazan absorption. This interference will result in misleading readings. 

Altogether, these findings indicate that the classical approach of MTT quantification using DOXO directly interferes in the metabolic activity measurement. Removing treatment media from the cell culture and exchanging to PBS results provides viable and clear results such as those demonstrated in a dose-response curve determination (Figure 2). 

We consider that the present work may contribute to the design of several in vitro cell culture experiments using colorimetric assays.

## References

[1] Peters W.P., Ross M., Vredenburgh J.J., Meisenberg B., Marks L.B., Winer E., Kurtzberg J., Bast R.C., Jones R., Shpall E. (1993). High-dose chemotherapy and autologous bone marrow support as consolidation after standard-dose adjuvant therapy for high-risk primary breast cancer. J. Clin. Oncol..

[2] Goodnow R.A., Davie C.P. (2017). DNA-Encoded Library Technology: A Brief Guide to Its Evolution and Impact on Drug Discovery. Annu. Rep. Med. Chem..

[3] Mosmann T. (1983). Rapid colorimetric assay for cellular growth and survival: Application to proliferation and cytotoxicity assays. J. Immunol. Methods.

[4] Berridge M.V., Herst P.M., Tan A.S. (2005). Tetrazolium dyes as tools in cell biology: New insights into their cellular reduction. Biotechnol. Annu. Rev..

[5] Twentyman P.R., Luscombe M. (1987). A study of some variables in a tetrazolium dye (MTT) based assay for cell growth and chemosensitivity. Br. J. Cancer.

[6] Riss T. (2014). Is Your MTT Assay Really the Best Choice?. Promega Corp..

